# Quantification metrics for telangiectasia using optical coherence tomography

**DOI:** 10.1038/s41598-022-05272-1

**Published:** 2022-02-02

**Authors:** Jillian L. Cardinell, Joel M. Ramjist, Chaoliang Chen, Weisong Shi, Nhu Q. Nguyen, Tiffany Yeretsian, Matthew Choi, David Chen, Dewi S. Clark, Anne Curtis, Helen Kim, Marie E. Faughnan, Victor X. D. Yang, the Brain Vascular Malformation Consortium HHT Investigator Group, Murali Chakinala, Marianne S. Clancy, Marie Faughnan, James R. Gossage, Katharine Henderson, Vivek Iyer, Raj S. Kasthuri, Helen Kim, Timo Krings, Michael T. Lawton, Doris Lin, Johannes Jurgen Mager, Douglas A. Marchuk, Justin P. McWilliams, Jamie McDonald, Ludmila Pawlikowska, Jeffrey Pollak, Felix Ratjen, Karen Swanson, Dilini Vethanayagam, Andrew J. White, Pearce Wilcox

**Affiliations:** 1 Deparment of Electrical, Computer, and Biomedical Engineering, Ryerson University, Toronto, ON Canada; 2 Department of Optical Engineering, Nanjing University of Science and Technology, Nanjing, Jiangsu China; 3 Physical Sciences Platform, Hurvitz Brain Sciences Research Program, Sunnybrook Research Institute, Toronto, ON Canada; 4 Toronto HHT Centre, Division of Respirology, Department of Medicine, St. Michael’s Hospital, University of Toronto, Toronto, ON Canada; 5 Division of Dermatology, University of Toronto, Toronto, ON Canada; 6 Department of Neurological Surgery, University of California, San Francisco, San Francisco, CA USA; 7 Li Ka Shing Knowledge Institute, St. Michael’s Hospital, Toronto, ON Canada; 8 Department of Surgery, Division of Neurosurgery, Sunnybrook Health Sciences Centre, University of Toronto, Toronto, ON Canada; 9 Washington University School of Medicine, St. Louis, MO USA; 10 HHT Foundation, Monkton, MD USA; 11 Division of Pulmonary, Critical Care, and Sleep Medicine, Augusta University, Augusta, GA USA; 12 Yale HHT Center, Yale University School of Medicine, New Haven, CT USA; 13 Division of Pulmonary and Critical Care Medicine, Mayo Clinic, Rochester, NY USA; 14 Division of Hematology and Oncology, Department of Medicine, University of North Carolina at Chapel Hill, Chapel Hill, NC USA; 15 Division of Neuroradiology, Department of Medical Imaging, Toronto Western Hospital, University of Toronto, Toronto, ON Canada; 16 Department of Neurosurgery, Barrow Neurological Institute, Phoenix, AZ USA; 17 Department of Neurosurgery, Johns Hopkins University School of Medicine, Baltimore, MD USA; 18 Department of Pulmonology, St. Antonius Hospital, VB Nieuwegein, The Netherlands; 19 Molecular Genetics and Microbiology, Duke University, Durham, NC USA; 20 Department of Radiology, David Geffen School of Medicine at UCLA, Los Angeles, CA USA; 21 Department of Radiology, University of Utah, Salt Lake City, UT USA; 22 Radiology and Biomedical Imaging, Yale University School of Medicine, New Haven, CT USA; 23 Division of Respiratory Medicine, Department of Pediatrics, Hospital for Sick Children, Toronto, ON Canada; 24 Pulmonology and Critical Care Medicine, Mayo Clinic, Scottsdale, AZ USA; 25 Department of Medicine, University of Alberta, Edmonton, AB Canada; 26 Edward Mallinkrodt Department of Pediatrics, Washington University School of Medicine, St. Louis Children’s Hospital, St. Louis, MO USA; 27 Respirologist, Pacific Lung Health Centre, St. Paul’s Hospital, Vancouver, BC Canada

**Keywords:** Biomarkers, Biomedical engineering, Health services, Quality of life, Therapeutics, Cardiovascular diseases, Medical imaging, Tomography

## Abstract

Hereditary hemorrhagic telangiectasia (HHT) is an autosomal dominant disorder that causes vascular malformations throughout the body. The most prevalent and accessible of these lesions are found throughout the skin and mucosa, and often rupture causing bleeding and anemia. A recent increase in potential HHT treatments have created a demand for quantitative metrics that can objectively measure the efficacy of new and developing treatments. We employ optical coherence tomography (OCT)—a high resolution, non-invasive imaging modality in a novel pipeline to image and quantitatively characterize dermal HHT lesion behavior over time or throughout the course of treatment. This study is aimed at detecting detailed morphological changes of dermal HHT lesions to understand the underlying dynamic processes of the disease. We present refined metrics tailored for HHT, developed from a pilot study using 3 HHT patients and 6 lesions over the course of multiple imaging dates, totalling to 26 lesion images. Preliminary results from these lesions are presented in this paper alongside representative OCT images. This study provides a new objective method to analyse and understand HHT lesions using a minimally invasive, accessible, cost-effective, and efficient imaging modality with quantitative metrics describing morphology and blood flow.

## Introduction

Hereditary Hemorrhagic Telangiectasia (HHT) is an autosomal dominant genetic disorder characterized by the presence of vascular malformations (VMs), with an estimated prevalence of 1 in 5000^1–3^. Smaller malformations, telangiectases, typically occur in the nasal, oral, and gastrointestinal mucosa, or on the skin, and result in acute or chronic bleeding and anemia. Larger lesions, arteriovenous malformations (AVMs), typically occur in the brain and lungs, with diffuse and confluent telangiectases typically affecting the liver^4,5^. More than 90% of adults with Hereditary Hemorrhagic Telangiectasia (HHT) develop chronic epistaxis, leading to significant morbidity, including anemia, emergency room visits, and impaired quality of life^6–10^. A recent explosion of potential new therapies for HHT, with most being antiangiogenic therapies, have the potential to rescue malformations and cure disease. Thus, there is a pressing need to develop outcome measures for trials in HHT that can relate lesional response to therapy. Our aim is to develop microimaging techniques for telangiectases in HHT as these lesions are frequent and accessible on the skin and mucosa, and should provide a window into response to new therapies.

Telangiectases in HHT are characterized by the loss of capillary bed between arterioles and venules, with dilated post-capillary venules^11,12^. This disordered interchange between arteriole and venules generates high perfusion pressure that can lead to chronic hemorrhage. Current knowledge of VM structure and morphology in HHT is based primarily on imaging of visceral AVMs with computed tomography, magnetic resonance imaging, and Doppler ultrasound^4,5^. There are only limited imaging studies of telangiectasia lesions, with one case study doing only visual analysis on dermal lesions and another study only performing ex vivo analysis, which lacks information about live morphology and responsiveness^12,13^. Few studies are focused on analysing therapeutic live responses of lesions, though our group has previously reported a detailed study of optical coherence tomography (OCT) applied to an HHT patient^14^. OCT is a non-invasive light interferometry imaging modality that can obtain high-resolution (< 10 µm), non-contrast, sub-surface (up to > 2 mm) images, and can provide the possibility of repetitive imaging studies to assess lesional treatment response in HHT trials^15,16^.

OCT Angiography (OCT-A) can delineate microvasculature by detecting blood flow through Doppler OCT (D-OCT) or Speckle Variance OCT (SV-OCT) methods^17,18^. SV-OCT is more resilient to different flow conditions making it a preferred to D-OCT^14,17,18^. SV-OCT methods are used by the VivoSight OCT System (Michelson Diagnostics Ltd., UK)—a Health Canada and FDA approved dermal OCT/OCT-A system^16^. Cheng et al. from our group had demonstrated the potential for OCT as an HHT imaging method by applying OCT flow mapping to HHT cutaneous lesions in vivo^14^. Their method was applied to the raw VivoSight data and their vascular images showed great potential, but were computationally expensive and noisy, and only were applied to one patient^14^. Shi et al*.* have since improved SV-OCT by enhancing delineation of dermal microvasculature, and increasing signal-to-noise ratio and spatial resolution with their differential standard deviation of log-scale intensity (DSDLI) algorithm^19^.

Other studies have seen success in using OCT-A to analyse dermal vascular pathologies, and have implemented quantitative metrics. Baran et al. used vascular OCT images to calculate area-based vessel density to use as a biomarker for acne lesion healing^20^. A detailed quantitative analysis of mouse ear vasculature was performed by Reif et al. using vessel density, box-counting fractal dimension, and vessel length fraction. Vessel length fraction modified vessel density using a skeletonized vessel image. Fractal dimension was also calculated on the skeletonized image^21^. Fractal dimension describes self-similarity and has been proven useful in studying pathological biological structures^22^.

A study using OCT methods on mouse cerebral vasculature used a more local analysis on skeletonized images to obtain a density measure to characterize angiogenesis^23^. Vakoc et al*.* used optical frequency domain imaging to image tumors in vivo on mice subjects and measured intratumor vessel length, tortuosity, volumetric fractal dimension, and mean vessel diameter to successfully track tumor progression through therapy^24^. A histological cancerous tumor study used numerical vessel density and vessel surface proportion within tumor hotspots as metrics^25^. Demidov et al. created radiation injuries in mice and imaged the area using SV-OCT. They were able to find significant differences between irradiated tissue and healthy tissue using vessel diameter measured from the SV-OCT images, which conform to the findings from histology-based methods^27^. These metrics have proven important in tracking angiogenesis and tumor progression, and can be applied to HHT lesions throughout a course of treatment; however, there is still a need to develop metrics tailored to progression monitoring of the unique morphology of telangiectases.

In this paper, we propose a pipeline for the processing and semi-automated structural feature quantification of telangiectases scanned using the VivoSight system^16^. Images were acquired during a pilot study for a topical beta-blocker treatment. This pilot allowed for the development and refinement of a set of quantitative metrics capable of analyzing lesion behaviour and response to therapies. These metrics were based off lesions with varying morphologies, and are capable of giving insight into progression and changes in the angiogenic process. Further, they can enable the development of personalized and responsively dynamic therapeutic plans to improve long term efficacy of treatments.

## Methods

### Patient selection

Three patients with definite clinical diagnosis of HHT were recruited, with at least 5 skin telangiectases available for imaging, for the Topical Antiangiogenic Pilot in HHT Trial (NCT01752049) from the Toronto HHT Centre—an HHT Centre of Excellence at St. Michael’s Hospital, University of Toronto. The study protocol was approved by the Unity Health Research Ethics Board of St. Michael’s Hospital. All patients provided written, informed consent to participate, and provided consent to publish results. All methods were performed in accordance with the relevant guidelines for human topical drug trials and imaging studies, as approved by the St. Michael's Hospital.

### Image acquisition protocol

The location of the lesion was photographed and labelled with a lesion number. The lesions were scanned during imaging appointments using the dynamic acquisition mode on the VivoSight, with appointments scheduled approximately once per month. Each patient attended 4–5 imaging sessions after a topical antibiotic treatment had begun.

**Figure 1:**
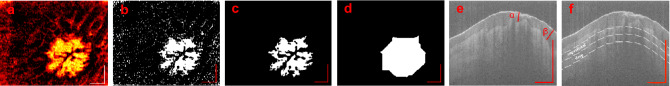
Image analysis pipeline for lesion quantification. (**a**) Enface image generated by DSDLI, (**b**) filtered and binarized image, (**c**) isolated lesion vasculature, (**d**) dilated and closed image, (**e**) structural slice image, and (**f**) shows the depths from where the deep and superficial images are constructed from. The axis in each image represents a 1 mm × 1 mm bar.

Once scanned on the VivoSight, raw data was extracted and used to construct angiographic images. Two enface vascular images (6 × 6 mm) were made using DSDLI (Fig. 1a). Superficial images were made from slices at approximately 330–510 µm below the skin surface, and deep images were made from slices 510–690 µm below the surface as shown in Fig. 1f. These images were displayed with a custom colourmap in order to maximize the contrast between vessel and tissue without losing vascular information. Structural enface images were also made at these depths. Four cross-sectional structural slice images showing the skin stratum at the lesion were generated from standard OCT extraction algorithms from each channel of the Vivosight (Fig. 1e).

Four lesions were included in the analysis for the first patient, 3 from the second patient, and 2 from the third. Lesions were excluded if there were less than 2 valid scans from separate imaging sessions. Valid scans required that both deep and superficial images could be acquired without major artefacts. A total of 28 valid lesion scans were collected across the 9 lesions.

### Image filtering

The vascular enface images from DSDLI (Fig. 1a), herein referred to as the original image, were filtered using compounded filtering techniques. First, the original image was passed through a 2D median filter with a 3 × 3 neighborhood to smooth out salt and pepper noise. A gaussian filter was then applied to the median-filtered image with a 3 × 3 kernel with a standard deviation of 3. This resultant image was then subtracted from the original image to create a difference image to reduce structural artefacts such as fingerprints. Another median filter with a large 8 × 8 neighborhood was applied to the original image and added to an amplified version of the difference image to restore and enhance the central region of the lesion that was reduced in the gaussian filtering. An optimal threshold of 0.6 was selected for binarization to further eliminate noise but retain vascular information (Fig. 1b). All processing was carried out in MATLAB.

### Metric calculation

The binary vascular enface images (Fig. 1b) were used to calculate Vascular Fraction (VF) which was defined as vascular pixels divided by total pixels in the image. This same binary image was also used to calculate the box fractal dimension using Fractalyse software (Fractalyse v2.4, 2006). Vascular binarized images were then area filtered to isolate lesion vasculature (Fig. 1c). The vertical and horizontal resolutions were used to calculate the Vascular Area (VA) of the lesion in mm^2^. To achieve approximate lesion area (LA), the isolated lesion vasculature was dilated and closed, and the area was calculated as previously described (Fig. 1d). A secondary metric was calculated as VA/LA to classify lesions as dense or diffuse and was termed the Vascular Area Lesional Value (VALVe). This process was done on both the superficial and the deep images.

The structural cross-sectional B-mode images were used to calculate epidermal fraction (EF). An observed relative thickness measure of the epidermis was taken over the lesion (α) and over healthy tissue (β), and the EF was calculated as α/β to classify lesions as deep or superficial (Fig. 1e). This unitless index allows for the relative measurement of lesion depth that is unaffected by any refractive index differences of patient tissues. This measurement was taken from each of the four channel cross-sectional images and the final EF was taken as the average.

### Approval for human experiments

All methods were performed in accordance with the guidelines for human topical drug trials and imaging studies, as approved by the St. Michael's Hospital research ethics board, the Unity Health Research Ethics Board.

### Consent to participate

All sites received approval from their local institutional review boards and all patients provided written informed consent for participation.

### Consent for publication

All sites received approval from their local institutional review boards and all patients provided written informed consent for participation and publication.

## Results

We analysed 6 lesions from 3 patients over multiple sessions, providing a total of 26 image stacks. We will be specifying the lesions by a patient number and lesion number in the notation P#L#. The lesions imaged in this study had a wide variety of morphologies and presented dynamic behaviour over imaging sessions. The range of metrics used allowed detection of changes in overall blood supply, lesion size, structure, and density. Several lesions from different patients are presented in this paper to demonstrate the purpose and versatility of the proposed metrics.

### Progression analysis

**Figure 2:**
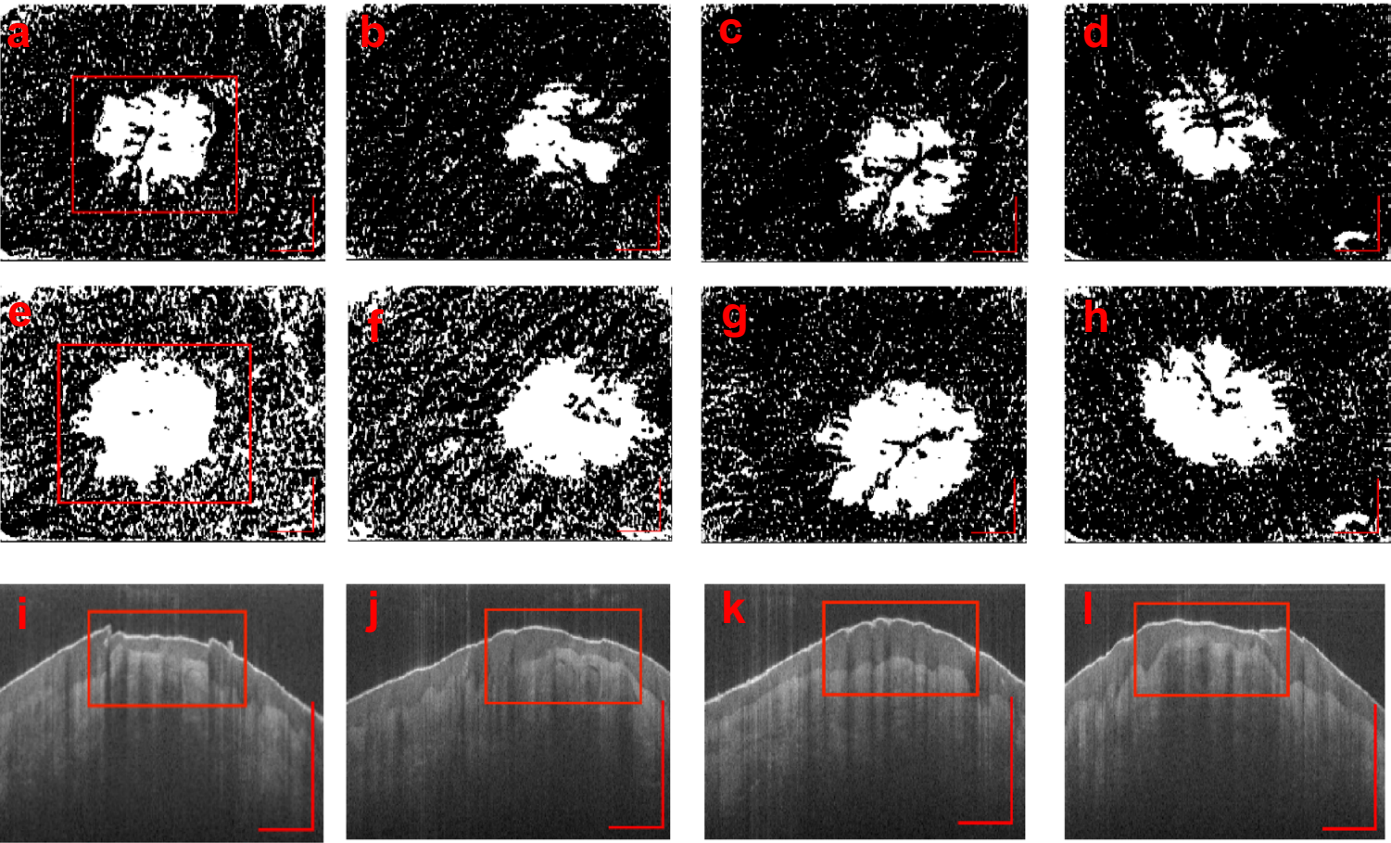
Progression at the superficial (**a**-**d**) and deep (**e**–**h**) slice-stacks, and the structural cross-sections (**i**-**l**) over the course of four imaging sessions P2L1. The axis in each image represents a 1 mm × 1 mm bar.

The images in Fig. 2a-d and e–h show the 6 × 6 mm filtered and binarized *en face* images from superficial and deep vascular ranges, respectively, of P2L1 over the course of imaging sessions. These figures reveal the densely concentrated network of the dilated and interconnected vasculature of the telangiectasia, as well as the supporting vessels. With the lesions shown at two depths, structural differences can be observed as the lesion protrudes into deeper dermal vascular networks. Figure 2i-l are the structural cross-sectional images generated from Channel 4 of the VivoSight that show the changes in the dermal and epidermal layers around the lesion. Uneven and bulging areas of the dermis are visible where the lesion swells into the epidermis.

Tables 1 and 2 show the changes in metrics from both the superficial and the deep layers, respectively, of the lesion shown in Fig. 2, P2L1, over the course of imaging sessions. From these metrics, we can see that the lesion exhibited an overall decrease in VF and VALVe in both superficial and deep layers; however, changes in LA and VLA were more variable. The third imaging session demonstrated a peak in most of the measurements, which could have been due to a warmer body temperature causing vascular dilation. The EF had also peaked at the 3rd imaging session. This measure corresponded with the increases in deep lesion vasculature enface metrics, potentially indicating a temporary shift in lesion centroid. FD showed a fairly consistent increase in both the superficial and the deep, demonstrating a decrease in vessel density and complexity. It should be noted that the lesions can be dynamic, being affected by both environmental (such as temperature) and external factors (including, possibly, the administered drug), leading to changes in the features at times.

**Table 1:** Changes in quantitative metrics calculated from the superficial images of P2L1.

Imaging session	VF	VA (mm^2^)	LA (mm^2^)	VALVe	FD
1	0.208	3.68	5.21	0.706	1.83
2	0.137	2.65	4.75	0.558	1.87
3	0.129	3.35	5.69	0.589	1.86
4	0.113	2.32	4.51	0.513	1.88

**Table 2:** Changes in quantitative metrics calculated from the deep images as well as the EF of P2L1.

Imaging session	VF	VA (mm^2^)	LA (mm^2^)	VALVe	FD	EF
1	0.402	5.35	6.84	0.782	1.83	0.25
2	0.345	4.49	6.51	0.689	1.85	0.30
3	0.250	5.63	7.67	0.735	1.84	0.71
4	0.234	5.25	7.08	0.741	1.84	0.24

### Characterization of lesion types

**Figure 3:**
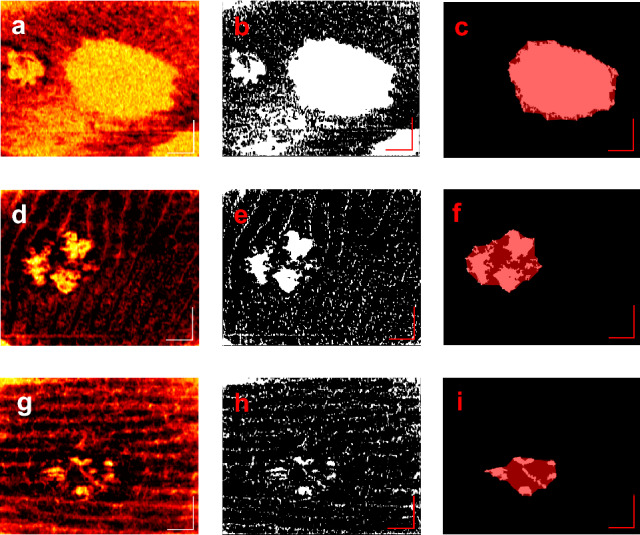
Three lesions from different patients demonstrating very different morphologies classifiable by VALVe. (**a**-**c**) is a deep slice example of a dense lesion, P2L2, with a VALVe = 0.903, (**d**-**f**) is a superficial slice example of an intermediate lesion, P1L1, with VALVe = 0.512, and (**g**-**i**) is a superficial slice example of a diffuse lesion, P3L1, with a VALVe = 0.290. The axis in each image represents a 1 mm × 1 mm bar.

The VALVe provided a detailed and unique insight on the lesion behavior and progression, and also served as a classification metric. Figure 3 shows three different lesion stack slices with different density classifications based on the VALVe. Figure 3a,d,g show the DSDLI images of each lesion and Fig. 3b,e,h show the filtered binary images. From these images, the differences in density are clearly visible; however, the VALVe provides a metric to quantitatively define more minute differences in densities. Figure 3c,f,i show an overlay of the lesion area in red on the isolated vascular area, providing a more definitive image of lesion density. A VALVe close to 1 indicated that the lesion was densely vascularized and likely very active like P2L2 shown in Fig. 3a, whereas VALVe closer to 0 indicated a very poorly vascularized and diffuse lesion as shown in Fig. 3g from P3L1. Density classification can provide insight on the complexity and robustness of the lesion being studied. These classifications are made at each depth for every lesion, which provides a more quantitative view of the nature of the vascular network of a lesion.

**Figure 4:**
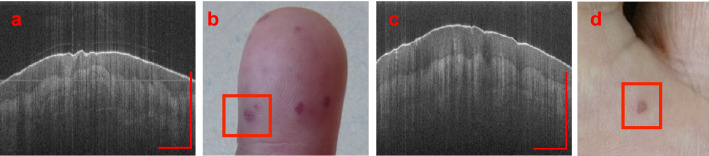
Two lesions from different patients demonstrating EFs and classifications. (**a**) is a superficial lesion, P2L2, with EF = 0.46 and (**b**) is the surface photograph of P2L2. (**c**) is a deep lesion, P1L1, with EF = 0.93 and (**d**) is the surface photograph of P1L1. The axis in each image represents a 1 mm × 1 mm bar.

Another metric also capable of lesion classification is the EF. Figure 4a is an example of a superficial lesion with the epidermis being very thin over the lesion relative to the healthy tissue. Figure 4b shows a deep lesion with the epidermis being relatively thick. This metric shows important information regarding the location and coverage of the lesion. Surface images of both the superficial and deep lesions are shown in Fig. 4b,d respectively. These photos further emphasize the need of OCT imaging and metrics as these images cannot show the depth and other physiological differences between different lesions.

### Overall quantitative changes

**Figure 5:**
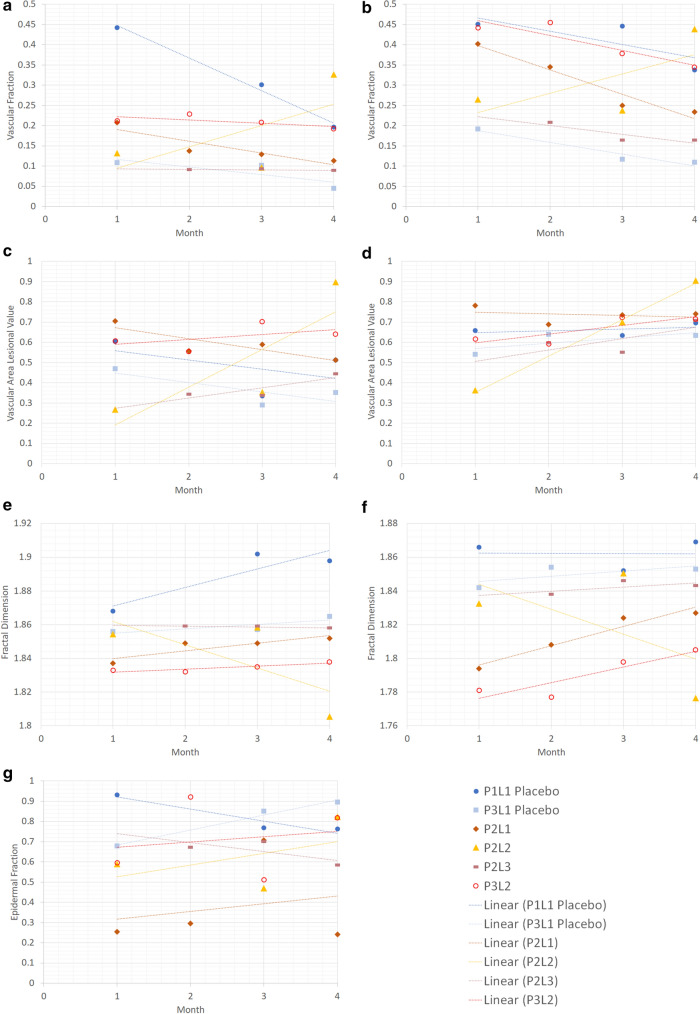
Graph of results for (**a**,**b**) vascular fraction, (**c**,**d**) VALVe, (**e**,**f**) Fractal Dimension, and (**g**) epidermal fraction, with (**a**,**c**,**e**) being superficial and (**b**,**d**,**f**) being deep, with (**h**) as a legend for all graphs.

The overall quantitative changes from the metrics of VF, VALVe, FD, and EF over the 4-month period are displayed in the graphs in Fig. 5. While images were acquired in all visits, not all images were suitable to the processing methods described above, and thus, some months do not have associated quantitative values. A generalized trendline has been used for each dataset to indicate any general changes observed throughout the imaging. These graphs demonstrate the capability of the metrics of tracking different morphological changes in lesions over time. By plotting the metrics of placebo and treated lesions, researchers can see the differences in dynamic behavior of lesions. Since this study did not control for confounding factors, these figures serve only to demonstrate the capacity of the metrics to track changes, and may not show the pure response to the treatment; however, this does demonstrate the ability to visualize differences between visits, and future studies that better control these confounding factors can allow for the rapid elucidation and identification of key trends that may demonstrate earlier positive or negative response to treatments.

### Environmental factors

**Figure 6:**
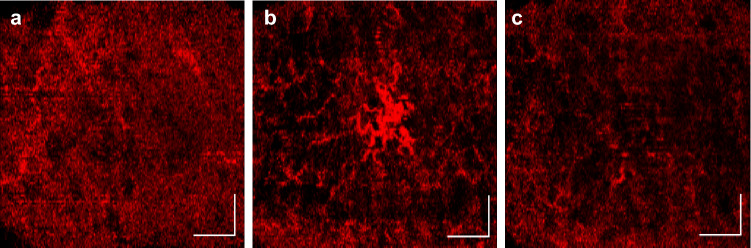
Vascular images directly from the VivoSight showing the same lesion (**a**) upon arrival, (**b**) after heating, (**c**) after cooling. The axis in each image represents a 1 mm × 1 mm bar.

A major confounding factor is temperature. The lesion shown in Fig. 6 demonstrates the impact of temperature on the lesion due to vasodilation, with the lesion being barely visible upon arrival for the visit (a) and after cooling (c), but clearly visible after the application of a warm cloth (b). Note that this lesion image was collected from a fourth patient, not included in the data presented in Fig. 5. This image was collected after the study was completed for proof of concept. This lesion appears completely different given different temperature conditions, and is practically undetectable from the VivoSight. This study took place across two seasons, increasing the likelihood that the temperature differences affected the presented results. Given these figures, it would be ideal to apply a warm wrap upon arrival before imaging to increase visibility and definition of the lesion and its surrounding structures in future studies.

## Discussion

This pilot study was an exploratory study to develop a protocol for acquiring, processing, analysing, and quantifying OCT images of cutaneous HHT lesions. As a result of this pilot, an integrated protocol can be defined which will optimize patient experience, usability, image quality and reliability, and measurement accuracy.

Current practice in analysing telangiectases relies mostly on visual inspection or histological imaging. In these practices, tracking and assessment is primarily qualitative, or is done by measuring approximate visible surface diameters^12,13^. In this paper, we have employed OCT-A and developed specific metrics that were able to track lesional changes and dynamic patterns in angiogenesis and blood flow, as well as structural changes in vivo. Dynamic angiographic metrics include VF—which was able to quantify blood flow of both the lesion and supporting vasculature, VLA—which provided isolated information on blood flow changes within the lesion area, LA—which showed information on the spread of the lesion and possibly indications of angiogenesis, and VALVe—which described lesion density. FD described size and complexity with a lower fractal dimension indicating lower complexity and decreased vasculature. EF indicated changes in the epidermis related to the lesion. Each metric provided important information on the lesion and its progression and were considered together to provide comprehensive analysis.

All lesions showed unique variations across the metrics indicating that meaningful morphological changes to lesion structures were detected over time. Although VF, VLA, and LA are all reliant on blood flow to the lesion, the changes over time in each of these metrics for a single lesion did not necessarily follow the same trend, which indicated the importance of collecting and analysing each of the metrics separately. Additionally, differences between superficial and deep metrics also were noted, revealing differences in lesion response depending on depth, and enforcing the need to measure metrics at different depths. Large scale, easily noticeable changes are easy to visually observe, but differences in the finer details may not be noticeable, providing the need for quantitative metrics capable of detecting the small changes in morphology and angiogenesis not visible to normal perception. The produced quantitative metrics show good potential for minute study of the underlying dynamic processes occurring below the surface.

Little major change or correlation could be determined from the quantitative metrics used above. Some lesions appeared to have little to no response, while others changed dramatically. As seen in Fig. 5, all lesions seemed to show a general decline in values related to the amount of blood flowing to vasculature; however, P3L2 seemed to show the opposite trend. It is difficult to determine if these changes are a consequence of treatment, natural changes to lesion morphology, or simply due to variability in external environment affecting the lesions. It is very possible that the treatment had no effect to the lesion and all of the noted changes in this study were due to variability in environmental factors and patient physiology. As well, given that the lesions themselves varied in size and morphology, some metrics are more effective than others at describing changes to morphology. Future studies with a larger treatment and control patient cohorts are needed in order to validate this pipeline for use as a clinical treatment assessment tool.

Given that vascular fraction evaluates the entire *en face* area imaged, changes to both the lesion and surrounding vasculature would have an impact on this metric. While used in analysis in other fields, this metric would be subject to error from global changes affecting the entire imaging area, and thus may not be representative of the dynamic processes in the lesion. Comparatively, VALVe isolates the vasculature in the lesion area, and would provide a more accurate analysis of how the lesion has changed over time. This is more clearly seen in Fig. 5a-d, where though a decrease in VF is observed in both deep and superficial, a very slight increase in the superficial VALVe and a variable trend for the deep VALVe is seen. Thus, this more direct approach could potentially give better insights into morphological changes compared to more traditional metrics like VF.

This analysis also provided valuable insight into potential sources for variability in a lesion, relating to both environmental factors and biological processes. Throughout the study, many of the lesions showed a peak in metrics late in treatment. Many of the imaging sessions started during a colder season and the peak in metrics was noticed in the imaging sessions completed in the warmer seasons. From this, we can predict that the dilation of the blood vessels was likely due to the change in temperature. This dilation can be seen in the sample data provided in Tables 1 and 2, as well as Fig. 2. The test performed with a single lesion under different temperature conditions, shown in Fig. 6, also demonstrates this phenomenon. The same lesion appears drastically different upon arrival, after heating, and after cooling, emphasizing the drastic need for temperature controls in future studies. A method of characterization and grouping of lesions can also be observed through both VALVe and EF. These metrics provide valuable insight related to the density and focus of the lesion, and could serve as useful tools in treatment planning. In this, we propose that lesions be classified as “dense” or “diffuse” using the VALVe measurement, and as “deep” or “superficial” according to EF. Figures 3 and 4 help to demonstrate the differences between these classifications. While a binary classification may be possible, a more graded scale may need to be employed; however, a larger dataset with more lesions should be studied in order to numerically assign values to these classifications. Nonetheless, these characterizations can help with treatment planning. For example, a deep, diffuse lesion may respond less well to topical therapies, or require adjustments to laser power to allow sufficient penetration for lesion ablation using laser therapies. There is also potential to apply these characterization metrics in diagnostic pipelines for younger patients or to diagnose uncertain lesions; however, more studies are necessary to determine the metric thresholds that could successfully differentiate a telangiectasia from other malformations. OCT can easily be adapted to and employed in a workflow to determine specific correlations not only for HHT, but beyond to other dermatological and lesional analysis which has previously been through qualitative visual inspection alone.

The process of image acquisition also revealed some systematic issues that could be improved upon. The primary issue was that the image acquisition is very slow, with a single scan requiring up to 3 min. This long acquisition period made the process very prone to motion artefacts. Many of the collected scans were rendered unsuitable for quantification due to these large motion artefacts. This long acquisition time also limited feasible imaging regions. Some lesions of interest found on other skin surfaces were not feasible for imaging as the patient and/or the technician were unable to maintain position for the full acquisition period. Additionally, the current probe system has a plastic offset that sets a focusing distance between the laser source and the skin surface. Depending on the skin location or consistency, epidermal thickness varies and the plastic offset needs to be changed. This can be tedious, with an optimal offset often unavailable. Further, holding the probe still against the skin will often induce pressure around the imaging region which can affect fine blood flow, potentially impacting the angiographic metrics observed. After imaging an area, there was often an indent on the skin surface from the plastic offset. Overall, system improvements including faster image acquisition and an adjustable probe standoff would increase the range of application of the current system. A completely non-contact probe would be a preferable solution, but may not be feasible without significant improvements to system acquisition, and compensatory mechanisms for bulk motion. Potentially, a real-time imaging system could alleviate much of this burden, but would require significant hardware improvements and optimization of current processing methods to be fully realized. Additionally, a previous study has indicated that structural OCT image quality may be slightly affected by skin tone; however, further investigation into OCT-A limitations with regard to skin tone need to be conducted^26^. Future improvements to acquisition methods to combat this issue would likely benefit this study as well as many others.

## Conclusion

In this pilot study, we have described a method for OCT acquisition, and outlined a post-processing pipeline that could potentially be used to provide quantitative analysis of HHT lesions. In this, we have reprocessed data taken from a clinical OCT system through a DSDLI algorithm with a series of standardized morphological operations in order to create an *en face* visualization of the complex vascular networks associated with HHT lesions. These images were then used to determine quantitative measures that could be of value for analyzing changes to these networks over time. While some attempts have been made at creating quantitative metrics for retinal OCT imaging, this is a significantly underdeveloped area for dermatological OCT. Thus, this provides a potentially generalized framework that could be employed not only for the study of HHT, but for other dermatological conditions where OCT imaging may be appropriate. As well, improvements to workflow and limitations to the current imaging system from experience are noted, with future work looking to overcome these limitations. Larger studies are needed to validate these measures for clinical trials in HHT.

## Data Availability

The data used in this study is property of St. Michael’s Li Ka Shing Knowledge Institute and Sunnybrook Research Institute. The datasets generated during and/or analyzed during the current study are available from the corresponding author on reasonable request.

## Code availability

The software used in this application is written in MATLAB R2019A and can be obtained upon reasonable request from the lead or corresponding author. The appropriate version of MATLAB can be downloaded from https://www.mathworks.com/products/new_products/release2019a.html. Additionally, Fractalyse v2.4 was used and can be downloaded at http://fractalyse.org/.
